# Improving the Efficacy of the Data Entry Process for Clinical Research With a Natural Language Processing–Driven Medical Information Extraction System: Quantitative Field Research

**DOI:** 10.2196/13331

**Published:** 2019-07-16

**Authors:** Jiang Han, Ken Chen, Lei Fang, Shaodian Zhang, Fei Wang, Handong Ma, Liebin Zhao, Shijian Liu

**Affiliations:** 1 Pediatric Translational Medicine Institute Shanghai Children’s Medical Center Shanghai Jiao Tong University School of Medicine Shanghai China; 2 School of Public Health Shanghai Jiao Tong University School of Medicine Shanghai China; 3 Synyi Research Shanghai China; 4 APEX Data and Knowledge Management Lab Shanghai Jiao Tong University Shanghai China; 5 Department of Healthcare Policy and Research Weill Cornell Medicine New York, NY United States; 6 Department of computer science Shanghai Jiao Tong University Shanghai China; 7 Child Health Advocacy Institute Shanghai Children’s Medical Center Shanghai Jiao Tong University School of Medicine Shanghai China

**Keywords:** electronic data capture, electric medical records, case report form, natural language processing, field research

## Abstract

**Background:**

The growing interest in observational trials using patient data from electronic medical records poses challenges to both efficiency and quality of clinical data collection and management. Even with the help of electronic data capture systems and electronic case report forms (eCRFs), the manual data entry process followed by chart review is still time consuming.

**Objective:**

To facilitate the data entry process, we developed a natural language processing–driven medical information extraction system (NLP-MIES) based on the i2b2 reference standard. We aimed to evaluate whether the NLP-MIES–based eCRF application could improve the accuracy and efficiency of the data entry process.

**Methods:**

We conducted a randomized and controlled field experiment, and 24 eligible participants were recruited (12 for the manual group and 12 for NLP-MIES–supported group). We simulated the real-world eCRF completion process using our system and compared the performance of data entry on two research topics, pediatric congenital heart disease and pneumonia.

**Results:**

For the congenital heart disease condition, the NLP-MIES–supported group increased accuracy by 15% (95% CI 4%-120%, *P*=.03) and reduced elapsed time by 33% (95% CI 22%-42%, *P*<.001) compared with the manual group. For the pneumonia condition, the NLP-MIES–supported group increased accuracy by 18% (95% CI 6%-32%, *P*=.008) and reduced elapsed time by 31% (95% CI 19%-41%, *P*<.001).

**Conclusions:**

Our system could improve both the accuracy and efficiency of the data entry process.

## Introduction

According to ClinicalTrials.gov [1], the number of clinical trials worldwide has increased exponentially in recent years. Clinicians and researchers use evidence from interventional and observational trials to determine the effectiveness of treatments or interventions. Interventional trials, such as randomized controlled trials, compare the efficacy of interventions under relatively ideal cohorts to get unbiased estimates of effects. However, reality is far more complicated, and these ideal cohorts limit generalizability of results obtained to broader patient populations and settings. Moreover, due to high expenses and the short research cycle, interventional trials could hardly provide evaluations of effectiveness and safety for large populations and long-term follow-ups. As supplements, many observational trials, such as retrospective cohort studies, cross-sectional studies, and real-world evidence studies, use patient historical data collected at the point of care to compare effectiveness and safety of treatments in clinical practice settings in nonexperimental ways. Such observational trials usually have larger cohort sizes and longer follow-up periods. Growing interest in using these approaches poses new challenges to effective and efficient collection of patient electronic medical records (EMRs).

Manual data entry based on paper-and-pen case report forms (CRFs) followed by chart review is the conventional way of clinical trial data collection. With the development of health care information technology, electronic data capture (EDC) systems, which accelerate the data collection process and assure data quality with real-time data entry, review, analysis, and verification [2], emerge as a timely solution that is in high demand. Driven by the prevalent use of EDC systems, CRFs gradually transitioned from paper to electronic forms [3]. Many studies have suggested that data entry using electronic CRF (eCRF) applications of EDC systems could achieve higher efficiency and accuracy at a lower cost than the conventional paper-and-pen approach [2,4-8]. However, neither EDC nor eCRF fundamentally changed the essential ways of how the data are collected. Especially for observational trials using patient data, researchers still need to manually transcribe the data one by one from EMRs. The data entry process takes time and becomes a significant efficiency bottleneck.

The 2018 guidance from the US Food and Drug Administration [9] emphasized the importance of interoperability between electronic health records (EHRs) and EDCs. It also promoted the idea of secondary use of source data at the time of care to prepopulate eCRFs without specific user efforts. The guidance focused more on the use of structured data, such as demographics, vital signs, and laboratory data, but little on the use of unstructured clinical narratives, which account for about 80% of the patient care information [10]. To achieve data interoperability for these unstructured narratives, many EDC systems created predesigned patient information templates including standardized documentation or forms for coded data entry in lieu of free text documentation to structuralize the medical records [11,12]. Clinicians record patient information under the guidance of these templates, and at the same time the system stored the coded data from templates for future analysis. Patient information templates can help data collection for research and patient care, integrate EDC and EMRs, and automatically prepopulate the eCRF. However, limitations of the templates were obvious. For clinicians, the one-size-fits-all templates restricted freedom of expression. For researchers, the predesigned data elements limited usability of the data in different research topics.

The development of natural language processing (NLP) technologies provides new potential for better secondary use of free unstructured EMR data. Informatics for integrating biology and the bedside (i2b2) has posed NLP challenges to extract information, including clinical finding, test, treatment, medication, clinical event, and time information, from clinical notes and discharge summaries [13-16] and promoted a series of commercial medical applications focusing on post hoc structuralization of medical records [17-19]. Nonetheless, as one of the main topics on secondary use of patient EMR, unstructured data collection based on NLP technology has not been well studied.

In order to fill in this gap, we developed an NLP-driven medical information extraction system (NLP-MIES) based on i2b2 reference standards for concept extraction, assertion, and relation classification. After manually constructing eCRFs and binding data elements using concepts from the Systematized Nomenclature of Medicine–Clinical Terms (SNOMED-CT) or the radiology-specific ontology (RadLex) developed by the Radiological Society of North America, our system can scan clinical notes and image diagnostic reports, find related medical concepts, and automatically prepopulate data elements with associated values. To further compare the accuracy and efficiency between manual data entry and NLP technology–supported data entry, we conducted a randomized and controlled field experiment. We created a mock-up eCRF application that enables users to review medical records and enter, modify, and verify the data prepopulated by NLP-MIES. We recruited clinicians and researchers to use the application to finish a certain amount of simply designed eCRFs in the limited time. Based on these designs, we simulated a real-world eCRF filling process and aimed to quantitatively evaluate how NLP technologies could improve efficacy of data collection of clinical research and identify potential problems that are not neglectable in future NLP-driven EDC design.

## Methods

### Natural Language Processing–Driven Medical Information Extraction System

**Figure 1:**
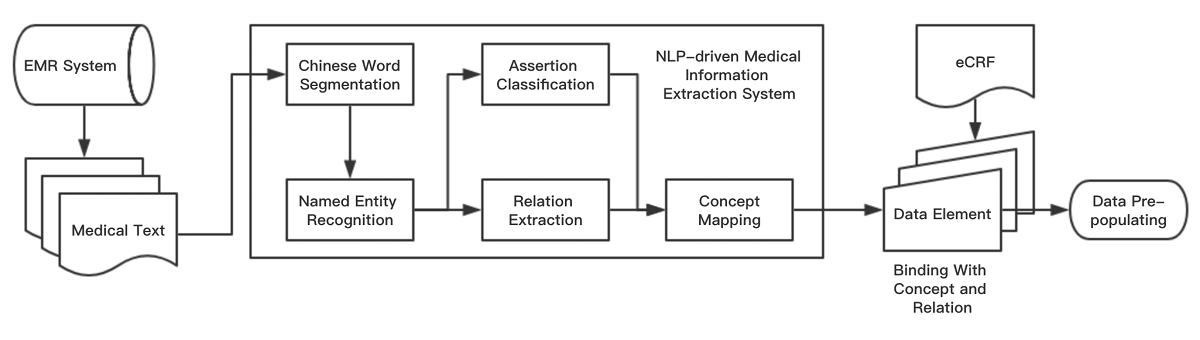
Workflow of the natural language processing–driven medical information extraction system. EMR: electronic medical record; NLP: natural language processing; eCRF: electronic case report form.

We leveraged the methods developed for the 2010 i2b2/Veterans Affairs (VA) challenge as the primary reference for Chinese medical NLP machine learning practices in NLP-MIES, which includes Chinese word segmentation, named entity recognition, assertion classification, and relation extraction [14,20-22]. On the basis of the predefined entities (medical problems, tests, treatments) and relation types (medical problems and treatments, medical problems and tests, medical problems and other medical problems) from the 2010 i2b2/VA challenge, in order to extract more information from medical records, we added four new entities (body structure, observable, qualifier, value) and four new types of relations (body structures and observables, medical problems and observables, observables and qualifiers, observables and values). After preprocessing by an associated value dimension algorithm [23], entities from medical texts can be rearranged according to their relations. We then adopted an improved longest common subsequence algorithm to map these aligned entities and relations into Chinese SNOMED-CT and RadLex concepts and synonyms [24]. Figure 1 shows the overall workflow of NLP-MIES.

### Electronic Clinical Research Form

**Figure 2:**
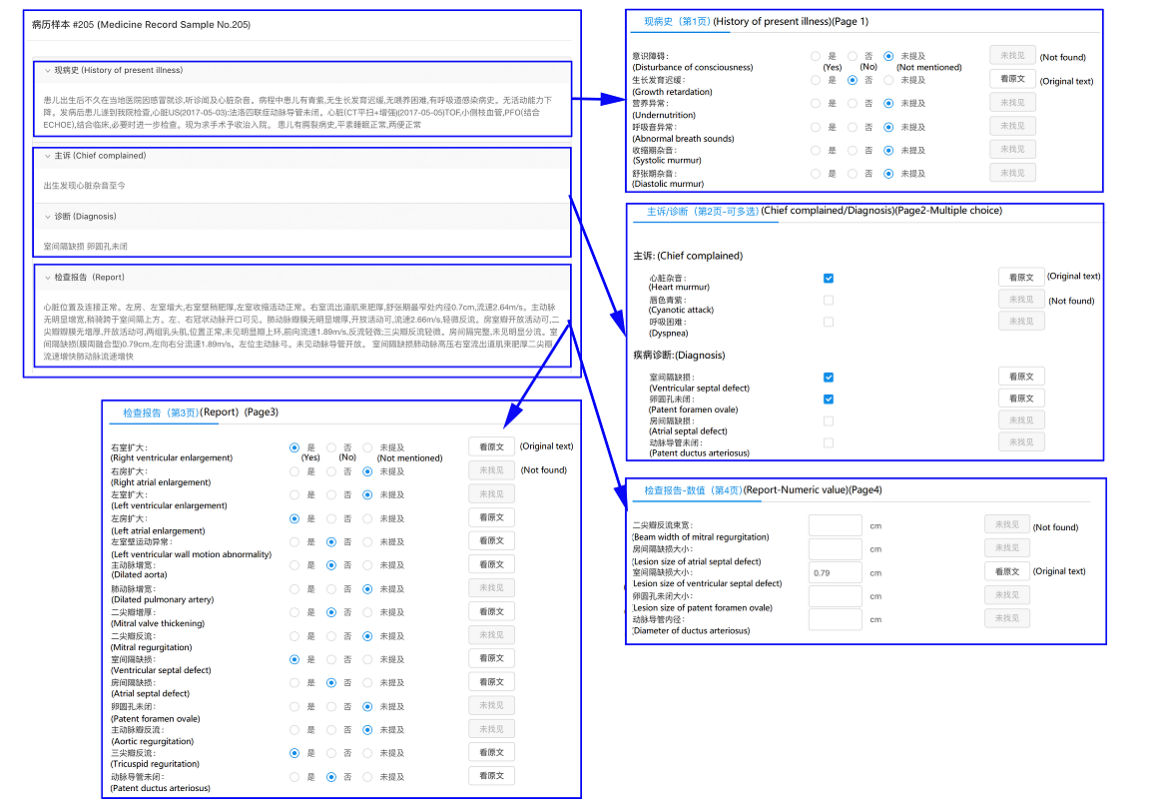
Electronic case report form design for congenital heart disease.

**Figure 3:**
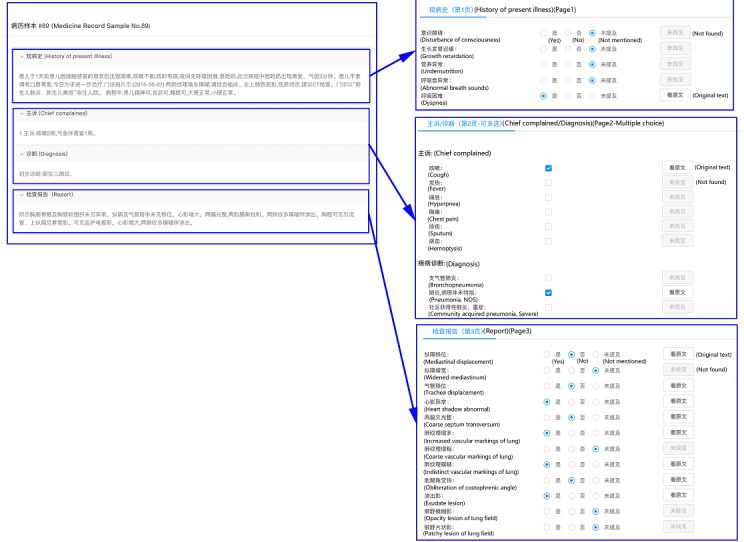
Electronic case report form design for pneumonia.

We constructed simple eCRFs for two disease conditions (pediatric congenital heart disease and pneumonia) to evaluate the efficacy of NLP-MIES. To make the eCRFs closer to the real ones, we invited clinical researchers from the departments of pediatric cardiothoracic surgery and pediatric respiratory medicine to help design the eCRFs. The types of CRF data elements include true-false (participant judges whether a certain condition or medical problem exists, doesn’t exist, or is not mentioned in a certain case and chooses the button accordingly—for example, patient had a disturbance of consciousness: true, false, or not mentioned); multiple choice (participant should click the button corresponding to one or more conditions or medical problems associated with a certain patient—for example, which of the following are the chief complaints of the patient: cardiac murmur, cyanosis, or dyspnea); and fill-in-the-blank (participant should enter the value for each data element—for example, the lesion size of ventricular septal defect is ___ cm). Figures 2 and 3 show examples of eCRF design.

We further divided the data element true-false into two parts based on where the elements should be retrieved from: admission records (true-false I) or imaging reports (true-false II). All data elements were bound with SNOMED-CT or RadLex concepts and relations, such as disturbance of consciousness (concept, medical problem, SNOMED-CT ID: 3006004), cardiac murmur (concept, medical problem, SNOMED-CT ID: 42842009), lesion size (concept, observable, SNOMED-CT ID: 246116008) of (relation, medical problems and observables) ventricular septal defect (concept, medical problem, RadLex ID: RID3277).

### Medical Text From the Electronic Medical Record System

For the congenital heart disease condition, we included admission records and ultrasonic cardiogram reports from pediatric patients aged 2 hours to 14 years with congenital heart disease (including atrial septal defect, ventricular septal defect, patent ductus arteriosus, patent foramen ovale, etc) attending the department of cardiothoracic surgery of Shanghai Children’s Medical Center from July 1, 2016, to July 1, 2017.

For the pneumonia condition, we included admission records and chest x-ray reports from pediatric patients aged 6 months to 14 years with pneumonia (including bronchopneumonia, viral pneumonia, bacterial pneumonia, mycoplasma pneumonia, lobar pneumonia, lobular pneumonia, etc) attending the department of respiratory medicine of Shanghai Children’s Medical Center from July 1, 2016, to July 1, 2017.

All medical texts were from the EMR system of Shanghai Children’s Medical Center and were de-identified. We randomly selected 60 patient cases for each condition. A total of 120 cases and 240 medical texts were included.

### System Functions and Human-Computer Interaction

**Figure 4:**
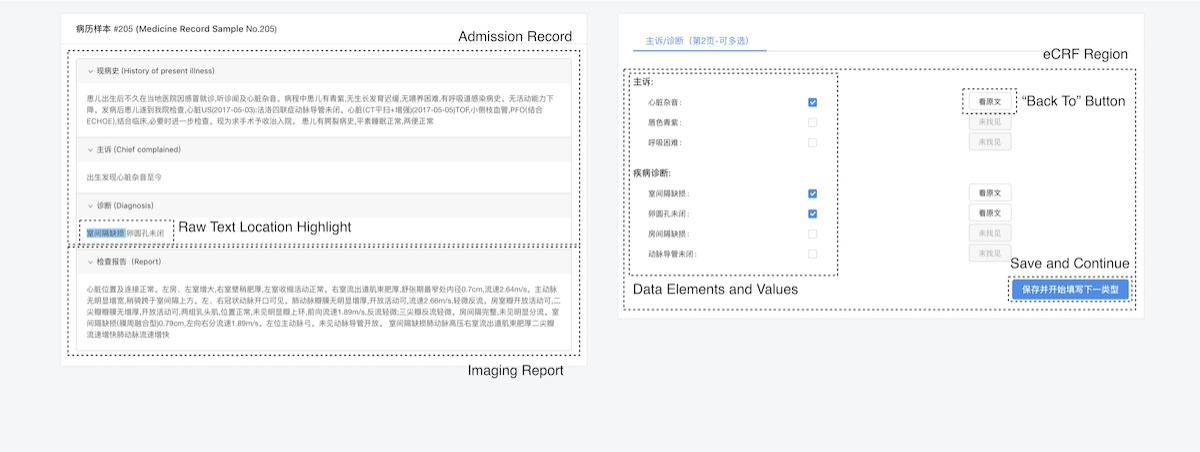
Graphic user interface for electronic case report form (eCRF) data entry.

We developed a graphical user interface for easy browsing of imported patient medical texts as shown in Figure 4. User can see imported admission records, imaging reports, and eCRFs on the screen. When NLP-MIES was enabled, our system automatically scanned the texts, found medical concepts mentioned in raw texts, identified assertion or value information, and prepopulated the data elements accordingly. Our system recorded the raw text location where each medical concept was extracted. When necessary, user could directly click the “back to” button to highlight the location for further data verification. Each eCRF was divided into three or four parts according to the types of data elements (Figures 2 and 3). During the experiment, the elapsed time for finishing each part was automatically recorded by system.

### Gold Standard

The ground truth results of eCRFs for all 120 cases were provided by three clinical researchers involved in the eCRF design. We used a two-step strategy to create our gold standard. First, two invited researchers independently extracted data from medical texts and populated eCRFs using an eCRF application but without the support of NLP-MIES. Our system automatically recorded the populated values and elapsed time for each data entry. Second, for pairs in which the two researchers did not have complete agreement, a third researcher resolved inconsistent data extraction between the two researchers.

### Study Design

We conducted a randomized and controlled field experiment at Shanghai Children’s Medical Center to evaluate whether the NLP-MIES group was more effective and efficient than the manual group in the data entry process of eCRF. Participants holding medical degrees, having clinical research experience, or working as clinicians were eligible for inclusion and recruited in this study. The study was approved by the Human Research Ethics Committees of Shanghai Children’s Medical Center. Written informed consent was obtained from all participants prior to randomization.

We randomly allocated the volunteers to two groups by using a completely randomized digital table:

- Manual group: participants should check the data elements in the eCRF, find related information in the medical text, and click or enter values accordingly.
- NLP-MIES–supported group: NLP-MIES prepopulated the data elements in the eCRF. Participants should check the data elements, find related information in the medical text, and verify or correct values accordingly.

Before the experiment, all participants were authorized and trained to use the system and eCRF-based data entry. We chose a relatively quiet place for the experiment to reduce the potential effect of other environmental factors. Each participant was provided with a laptop and asked to complete all cases from 2:00 pm to 5:00 pm. Participants failing to complete the eCRFs in that time frame were excluded from the data analysis. The order of the 120 cases was randomly shuffled for each participant.

### Outcomes and Statistical Analysis

We calculated average accuracy and elapsed time for each participant to finish all assigned eCRFs and compared the differences between the manual and NLP-MIES–supported group. To further analyze data entry errors made by participants under the support of NLP-MIES, we performed a post hoc error analysis for the results provided by NLP-MIES–supported group. We calculated the percentages of two types of data entry errors: error with modification and error without modification. We defined an error with modification as a data entry error made when a participant incorrectly modified a prepopulated result and an error without modification as a data entry error made when a participant kept an incorrect prepopulated result.

Educational and psychological studies have indicated that the distributions of the measurements of how many points participants could get in a certain test and how much time it would take a participant to respond to a certain stimulus (reaction time) were right-skewed [25-27]. Thus, we expected the data for each participant’s average accuracy and elapsed time for finishing eCRFs would not be normally distributed and described them using their median and interquartile range. To evaluate the differences between groups, we made a logarithmic transformation of the data and performed independent group *t* tests with SAS 9.2 (SAS Institute) software. *P* value, logarithmic mean difference (MD), ratio of change in geometric mean (exponential of logarithmic mean difference), and corresponding 95% confidence interval were calculated [28]. We considered two-sided *P* values <.05 as statistically significant.

## Results

### Participant Characteristics

We recruited a total of 24 eligible participants, 12 for the manual group and 12 for the NLP-MIES–supported group. All the participants successfully completed the eCRFs within the required time. The mean age of participants was 24.66 (SD 2.30) years (manual group 24.70 [SD 2.47] years, NLP-MIES group 24.48 [SD 2.36] years; *P*=.73); 33% (8/24) of participants were men and 67% (16/24) were women. There were no significant differences between the characteristics of the participants in the two groups.

The overall interoperator consistency rate was 96.85% (1627/1680) for the congenital heart disease condition and 94.82% (1081/1440) for the pneumonia condition (Multimedia Appendix 1).

### Accuracy

The overall average accuracy for the congenital heart disease and pneumonia eCRFs was significantly higher in the NLP-MIES–supported group than the manual group (congenital heart disease, *P*=.03; pneumonia, *P*=.008; Table 1). For the congenital heart disease eCRFs, the logarithmic MD of average accuracy between groups was 0.14 (95% CI 0.03-0.25), corresponding to an increase of 15% (95% CI 4%-120%) in geometric mean. Similarly, for the pneumonia eCRFs, the logarithmic MD was 0.17 (95% CI 0.06-0.28), corresponding to an increase of 18% (95% CI 6%-32%) in geometric mean. Comparing by types of data elements, the average accuracy was significantly higher in the NLP-MIES–supported group for all types except true-false II and fill-in-the-blank on the congenital heart disease eCRFs. The average accuracy of NLP-MIES prepopulation was slightly higher than median average accuracy of the manual group but lower than that of the NLP-MIES–supported group for most data element types.

### Elapsed Time

The overall average time elapsed for congenital heart disease and pneumonia eCRFs was significantly lower in the NLP-MIES–supported group than the manual group (congenital heart disease, *P*<.001; pneumonia, *P*<.001; Table 2). For the congenital heart disease eCRFs, the logarithmic MD of average time elapsed was –0.40 (95% CI –0.55 to –0.25), corresponding to a reduction of 33% (95% CI 22% to 42%) in geometric mean. For the pneumonia eCRFs, the logarithmic MD was –0.37 (95% CI –0.53 to –0.21), corresponding to a reduction of 31% (95% CI 19% to 41%) in geometric mean. Comparing by types of data elements, the average elapsed time was significantly lower in the NLP-MIES–supported group for all types.

### Error Analysis

Post hoc error analysis showed that errors without modification held the majority of error cases in all types of data elements (Table 3), and the overall percentage of errors without modification was almost 2.5 time higher than the percentage of errors with modification.

**Table 1:** Average accuracy for electronic case report form data entry.

Type of disease and data element		NLP^a^ only	Manual group (median, IQR^b^)	NLP-MIES^c^ group (median, IQR)	Logarithmic mean difference (95% CI)	Ratio of change in geometric mean (95% CI)	*P* value
**Congenital heart disease**							
	True-false I^d^	97.50	79.17 (66.74, 84.17)	96.81 (95.69, 97.29)	0.41 (0.04 to 0.79)	1.51 (1.03 to 2.20)	.04
	True-false II^e^	92.00	95.39 (92.67, 95.89)	97.78 (97.19, 98.44)	0.21 (–0.01 to 0.10)	1.10 (0.99 to 1.24)	.10
	Multiple choice	89.33	82.80 (73.13, 85.83)	95.00 (94.58, 97.42)	0.29 (0.10 to 0.49)	1.34 (1.10 to 1.63)	.009
	Fill-in-the-blank	94.17	96.33 (95.25, 97.00)	97.00 (95.83, 97.42)	0.01 (–0.01 to 0.02)	1.01 (0.99 to 1.02)	.22
	Overall	92.77	90.42 (87.75, 92.68)	97.17 (96.83, 97.44)	0.14 (0.03 to 0.25)	1.15 (1.04 to 2.20)	.03
**Pneumonia**							
	True-false I	88.00	70.83 (65.25, 77.75)	88.17 (87.25, 89.00)	0.30 (0.11 to 0.50)	1.35 (1.11 to 1.65)	.009^f^
	True-false II	94.44	91.25 (88.26, 93.78)	95.83 (95.21, 96.81)	0.11 (0.01 to 0.21)	1.12 (1.01 to 1.23)	.04
	Multiple choice	80.83	67.50 (50.21, 72.50)	81.25 (77.92, 85.00)	0.33 (0.14 to 0.52)	1.39 (1.15 to 1.68)	.003^f^
	Overall	84.15	84.21 (80.53, 86.23)	92.19 (91.49, 93.20)	0.17 (0.06 to 0.28)	1.18 (1.06 to 1.32)	.008

^a^NLP: natural language processing.

^b^IQR: interquartile range.

^c^NLP-MIES: NLP-driven medical information extraction system.

^d^True-false I: data elements retrieved from admissions records.

^e^True-false II: data elements retrieved from imaging reports (ultrasonic cardiogram or chest x-ray).

^f^Independent group *t* test.

**Table 2:** Average elapsed time for electronic case report form data entry.

Type of disease and data element		Manual group seconds (median, IQR^a^)	NLP-MIES^b^ group seconds (median, IQR)	Logarithmic mean difference (95% CI)	Ratio of change in geometric mean (95% CI)	*P* value
**Congenital heart disease**						
	True-false I^c^	26.43 (21.43, 30.24)	13.84 (11.83, 16.06)	–0.71 (–1.02 to –0.39)	0.49 (0.36 to 0.68)	<.001
	True-false II^d^	49.48 (43.08, 51.44)	35.47 (31.34, 38.63)	–0.29 (–0.46 to –0.11)	0.75 (0.63 to 0.89)	.003
	Multiple choice	9.70 (10.61, 12.29)	7.34 (7.47, 8.55)	–0.36 (–0.53 to –0.19)	0.70 (0.59 to 0.82)	<.001
	Fill-in-the-blank	18.41 (17.35, 19.60)	12.38 (11.38, 14.70)	–0.34 (–0.50 to –0.17)	0.71 (0.60 to 0.84)	<.001
	Overall	103.79 (94.59, 109.39)	69.73 (60.91, 79.66)	–0.40 (–0.55 to –0.25)	0.67 (0.58 to 0.78)	<.001
**Pneumonia**						
	True-false I	28.71 (25.61, 32.61)	15.82 (14.36, 16.88)	–0.64 (–0.97 to –0.30)	0.53 (0.38 to 0.74)	.001
	True-false II	31.59 (28.29, 32.49)	25.22 (22.07, 28.80)	–0.19 (–0.35 to –0.03)	0.83 (0.71 to 0.97)	.02
	Multiple choice	11.02 (10.65, 12.05)	8.61 (8.05, 9.25)	–0.33 (–0.51 to –0.15)	0.72 (0.60 to 0.86)	.001
	Overall	73.28 (65.80, 74.47)	49.42 (44.33, 53.88)	–0.37 (–0.53 to –0.21)	0.69 (0.59 to 0.81)	<.001

^a^IQR: interquartile range.

^b^NLP-MIES: NLP-driven medical information extraction system.

^c^True-false I: data elements retrieved from admissions records.

^d^True-false II: data elements retrieved from imaging reports (ultrasonic cardiogram or chest x-ray).

**Table 3:** Error analysis for natural language processing–driven medical information extraction system–supported data entry.

Types	Errors, n (%)			
	True-false (n=1167)	Multiple choice (n=439)	Fill-in-the-blank (n=121)	Total (N=1727)
Errors with modification	325 (27.85)	158 (36.00)	16 (13.22)	499 (28.89)
Errors without modification	842 (72.15)	281 (64.01)	105 (86.78)	1228 (71.11)

## Discussion

### Principal Findings

In this field experiment, we created a mock-up eCRF application with NLP-supported data entry and simulated a real-world eCRF completion process. Results showed a consistent trend across all eCRF topics and data element types indicating NLP-MIES could significantly improve the accuracy and efficiency of data entry. In quantitative evaluation, data entry under the support of NLP-MIES could increase accuracy by approximately (relative change in geometric mean is similar to the change in arithmetic mean) [29] 15% to 18% and reduce elapsed time by one-third.

Many potential factors could contribute to the increased accuracy and efficiency of NLP-MIES–aided data entry. First, we considered NLP-MIES–aided data entry as in essence a process of double-checking—an NLP-MIES check followed by a manual check. In clinical practice, double-checking is a widely used and trusted approach that could significantly reduce medical errors [30,31]. Second, we tried several ways to establish participant trust in NLP-MIES: ensuring NLP-MIES entry accuracy (not worse or even better than manual entry), providing better interpretability (one-click back to raw text), and simplifying system interaction [28]. Third, the overall time elapsed for the manual group was about 50% more than the NLP-MIES–supported group. In our study, higher accuracy was achieved for pneumonia cases than congenital heart disease cases; it may be that extracted information on congenital heart disease cases was more complicated than that of pneumonia cases.

In our post hoc error analysis, we considered errors with modification as cognitive errors. Participants made cognitive errors because they failed to find correct answers (due to limitation of knowledge or lack of training) even though they noticed prepopulated answers were wrong. We considered most errors without modification as commission errors. Participants made commission errors because they followed the prepopulated answers that were incorrect. The result of error analysis indicated that commission errors dominated the data entry quality under the support of NLP-MIES. Overreliance could be a key factor for commission errors and as a side effect of participant trust in NLP-MIES [29]. One possible solution to this problem could be to use NLP-MIES as an independent investigator. In real-world clinical research data management, at least two investigators independently enter data for each case to reduce commission errors and then submit the entries to the clinical research associates (CRAs). The CRAs review and verify the entries to ensure data completeness and quality [30]. In our scenario, the NLP system could act as an independent investigator and provide data entry directly to CRAs rather than prepopulate data for other investigators, and CRAs could make final decisions based on both NLP-MIES–supported and manual entries.

### Strengths and Limitations

As far as we know, this is the first study, especially in Chinese language settings, that quantitatively evaluated how NLP technologies could improve the efficiency and efficacy of data collection of clinical research. We believe NLP technologies would be a vital link in the great chain of data exchange between EHRs and EDC. It can potentially extract and transform data from medical text in real time and pose fewer restrictions on clinician freedom of expressions and workflows. In addition, our mock-up NLP-driven eCRF application provided graphical user interface for easy browsing and validation of source text data and data entries to ensure data quality. We believe that the results of our study can provide guidance of future research and development of NLP-driven EDC systems as well as the integration of EDC and EMR systems.

Although the results of our field experiment demonstrated beneficial outcomes for NLP-MIES–supported data entry, there were limitations. First, we did not evaluate the efficacy of NLP-MIES under different prepopulation. Early research has indicated that improving accuracy of the automation system itself may not necessarily improve the performance of human-computer collaboration [31]. Moreover, some studies suggest that automation systems with low accuracy can affect human-computer collaboration and trust [32]. Second, there might be significant differences between our eCRFs and real-world CRFs in contents and types of data elements. Thus, it is inappropriate to extrapolate our quantitative results to real-world settings. Third, since NLP-MIES was designed for Chinese medical records and tested in Chinese eCRFs only, the efficiency of this methodology based on the i2b2 reference standard needs further evaluation in other languages.

### Conclusions

In this study, we developed an NLP-driven medical information extraction system based on i2b2 reference standards to facilitate the data entry process of eCRFs for clinical research. We conducted a randomized and controlled field experiment to simulate a real-world data entry process and evaluated the efficacy of our system. The results of our study showed NLP-MIES could significantly improve the accuracy and efficiency of data entry.

## Appendix

Multimedia Appendix 1 Interoperator agreement and elapsed time for each electronic case report form topic.

## Abbreviations

CRA: clinical research associate
CRF: case report form
eCRF: electronic case report form
EDC: electronic data capture
EHR: electronic health record
EMR: electronic medical record
i2b2: informatics for integrating biology and the bedside
IQR: interquartile range
MD: mean difference
NLP: natural language processing
NLP-MIES: natural language processing–driven medical information extraction system
SNOMED-CT: Systematized Nomenclature of Medicine–Clinical Terms
VA: Veterans Affairs
