# Supplementary material for: Improving the Efficacy of the Data Entry Process for Clinical Research With a Natural Language Processing–Driven Medical Information Extraction System: Quantitative Field Research
Source: JMIR Med Inform. 2019 Jul 16;7(3):e13331. doi: 10.2196/13331 (PMC6672807; doi:10.2196/13331)
Supplement: Multimedia Appendix 1 [file medinform_v7i3e13331_app1.pdf]

Appendix Table 1. Inter-operator agreement and consumed time for each eCRF topic.

eCRF topic	Data element type	Average consumed time (Median, IQR, unit: second)	Data element name (Chinese)	Data element name (English)	Consistence rate[a] (unit: %)
**Congenital heart disease**	True-false I	23.32 (20.57, 26.70)	意识障碍	Disturbance of consciousness	98 (59/60)
			生长发育迟缓	Growth retardation	98 (59/60)
			营养异常	Undernutrition	98 (59/60)
			呼吸音异常	Abnormal breath sounds	98 (59/60)
			收缩期杂音	Systolic murmur	100 (60/60)
			舒张期杂音	Diastolic murmur	97 (58/60)
	Choice	9.53 (7.58, 10.48)	主诉	Chief complaint	88 (53/60)
			疾病诊断	Diagnosis	92 (55/60)
	True-false II	39.64 (37.08, 42.09)	右室扩大	Right ventricular enlargement	90 (54/60)
			右房扩大	Right atrial enlargement	93 (56/60)
			左室扩大	Left ventricular enlargement	97 (58/60)
			左房扩大	Left atrial enlargement	98 (59/60)
			左室壁运动异常	Left ventricular wall motion abnormality	97 (58/60)

		主动脉增宽	Dilated aorta	98 (59/60)
		肺动脉增宽	Dilated pulmonary artery	100 (60/60)
		二尖瓣增厚	Mitral valve thickening	100 (60/60)
		二尖瓣反流	Mitral regurgitation	100 (60/60)
		室间隔缺损	Ventricular septal defect	97 (58/60)
		房间隔缺损	Atrial septal defect	98 (59/60)
		卵圆孔未闭	Patent foramen ovale	93 (56/60)
		主动脉瓣反流	Aortic regurgitation	97 (58/60)
		三尖瓣反流	Tricuspid reguritation	97 (58/60)
		动脉导管未闭	Patent ductus arteriosus	98 (59/60)
Fill-in-the-blank	12.87 (11.97, 15.30)	二尖瓣反流束宽	Beam width of mitral regurgitation	98 (59/60)
		房间隔缺损大小	Lesion size of atrial septal defect	95 (57/60)
		室间隔缺损大小	Lesion size of ventricular septal defect	98 (59/60)
		卵圆孔未闭大小	Lesion size of patent foramen ovale	98 (59/60)

			动脉导管内径	Diameter of ductus arteriosus	98 (59/60)
	Overall	88.69 (82.13, 98.01)	/	/	97(1627/1680)
**Pneumonia**	True-false I	21.03 (19.03, 24.65)	意识障碍	Disturbance of consciousness	97 (58/60)
			生长发育迟缓	Growth retardation	95 (57/60)
			营养异常	Undernutrition	92 (55/60)
			呼吸音异常	Abnormal breath sounds	92 (55/60)
			呼吸困难	Dyspnea	97 (58/60)
	Choice	7.51 (6.67, 8.27)	主诉	Chief complaint	90 (54/60)
			疾病诊断	Diagnosis	92 (55/60)
	True-false II	20.85 (18.87, 23.43)	纵膈移位	Mediastinal displacement	98 (59/60)
			纵膈增宽	Widened mediastinum	93 (56/60)
			气管位移	Trachea displacement	93 (56/60)
			心影异常	Heart shadow abnormal	92 (55/60)
			两膈欠光整	Coarse septum transversum	98 (59/60)
			肺纹理增多	Increased vascular markings of lung	98 (59/60)
			肺纹理增粗	Coarse vascular markings of lung	98 (59/60)

		肺纹理模糊	Indistinct vascular markings of lung	95 (57/60)
		肋膈角变钝	Obliteration of costophrenic angle	95 (57/60)
		渗出影	Exudate lesion	97 (58/60)
		肺野模糊影	Opacity lesion of lung field	95 (57/61)
		肺野片状影	Patchy lesion of lung field	95 (57/60)
Overall	50.86 (48.23, 52.96)	/	/	95(1081/1140)

[a] Consistence rate: the number of data elements in which the two researchers did not have complete agreement divided by the total number of data elements.
